# The metabolic regimes of 356 rivers in the United States

**DOI:** 10.1038/sdata.2018.292

**Published:** 2018-12-11

**Authors:** Alison P. Appling, Jordan S. Read, Luke A. Winslow, Maite Arroita, Emily S. Bernhardt, Natalie A. Griffiths, Robert O. Hall, Judson W. Harvey, James B. Heffernan, Emily H. Stanley, Edward G. Stets, Charles B. Yackulic

**Affiliations:** 1 U.S. Geological Survey, University Park, PA 16802, USA; 2 U.S. Geological Survey, Middleton, WI 53562, USA; 3 Department of Biological Sciences, Rensselaer Polytechnic Institute, Troy, NY 12180 USA; 4 Flathead Lake Biological Station, University of Montana, Polson, MT 59860 USA; 5 Department of Plant Biology and Ecology, University of the Basque Country, Bilbao 48080, Spain; 6 Department of Biology, Duke University, Durham, NC 27708, USA; 7 Climate Change Science Institute and Environmental Sciences Division, Oak Ridge National Laboratory, Oak Ridge, TN 37831, USA; 8 U.S. Geological Survey, Reston, VA 20192, USA; 9 Nicholas School of the Environment, Duke University, Durham, NC 27708, USA; 10 Center for Limnology, University of Wisconsin, Madison, WI 53706, USA; 11 U.S. Geological Survey, Boulder, CO 80309, USA; 12 U.S. Geological Survey, Flagstaff, AZ 86001, USA

**Keywords:** Carbon cycle, Ecosystem ecology, Freshwater ecology, Limnology

## Abstract

A national-scale quantification of metabolic energy flow in streams and rivers can improve understanding of the temporal dynamics of in-stream activity, links between energy cycling and ecosystem services, and the effects of human activities on aquatic metabolism. The two dominant terms in aquatic metabolism, gross primary production (GPP) and aerobic respiration (ER), have recently become practical to estimate for many sites due to improved modeling approaches and the availability of requisite model inputs in public datasets. We assembled inputs from the U.S. Geological Survey and National Aeronautics and Space Administration for October 2007 to January 2017. We then ran models to estimate daily GPP, ER, and the gas exchange rate coefficient for 356 streams and rivers across the continental United States. We also gathered potential explanatory variables and spatial information for cross-referencing this dataset with other datasets of watershed characteristics. This dataset offers a first national assessment of many-day time series of metabolic rates for up to 9 years per site, with a total of 490,907 site-days of estimates.

## Background & Summary

Primary production and aerobic respiration dominate metabolic energy flow and organic matter processing in stream ecosystems^1,2^. Stream metabolic responses to nutrient loading, anthropogenic modification, and natural disturbance regimes are likely to result in changes to stream processing of energy, carbon, and nutrients. Long-term time series (months to years) are particularly valuable because of the variability in stream metabolism at multiple temporal scales. Studies using such data have revealed the high sensitivity of annual in-stream production to the interaction of seasons and storms^3^, the stimulation of ecosystem respiration by polluted waters^4^, the potential for floodplain restoration to increase the resilience of stream metabolism to physical disturbance^5^, the correlation between winter precipitation and and spring heterotrophy in alpine streams^6^, and the constraining effect of turbidity on primary productivity in large rivers^7^. However, there is a pressing need to broaden our understanding of the controls on stream metabolism using standardized modeling approaches across multiple watersheds, ecoregions, climatic zones, and land use types^8^.

Daily estimates of reach-averaged gross primary production (GPP) and aerobic ecosystem respiration (ER) can be made from subdaily observations of the dissolved oxygen concentration, water temperature, average upstream depth, air pressure, and photosynthetically active radiation at a single location in the channel^9^. Metabolism modeling methods have advanced in recent years, in part due to the increasing speed and processing power of modern personal computers. Key advances have included estimation of gas exchange simultaneously with metabolism^10^, the use of Bayesian priors to incorporate field measurements or other external sources of information in the estimation procedure^11,12^, the use of state space models to accommodate multiple error sources^13,14^, and the use of Bayesian hierarchical modeling to pool information about gas exchange rate coefficients across many days of time series data^14–16^. A recently developed metabolism software package, streamMetabolizer^14^, integrates all of these advances to estimate metabolism.

Although metabolism has historically been studied at a small number of sites or using a small number of days per study, large-scale monitoring and modeling programs have now made it possible to estimate metabolism for a much larger number of sites. The U.S. Geological Surveyʼs (USGS) National Water Information System (NWIS) is a national database of time series observations of water quality and quantity for thousands of sites in the United States. NWIS contains several variables that are useful in estimating metabolism by a single-station open-channel approach^9^, including dissolved oxygen concentration, water temperature, and discharge. Two other useful variables, air pressure and downwelling shortwave radiation, are available through the National Aeronautics and Space Administrationʼs (NASA) Land Data Assimilation System (North American: NLDAS; Global: GLDAS), which synthesizes observations and model predictions into large-scale gridded datasets of climate and hydrology. These national databases made our national-scale analysis feasible through their well-documented data collection and modeling methods, consistent data formatting, and public accessibility.

In this data release we compile the data inputs and metabolism model outputs for 356 sites across the United States, with the resulting estimates ranging from 61 days to 9 years per site. Our objectives in creating this dataset are threefold: (1) to greatly expand the number of long-term metabolism time series in the literature by providing estimates of metabolism for 356 federally monitored sites, (2) to provide a data framework for experimentation with input datasets (several alternatives are provided for variables including light, barometric pressure, and stream depth) and models (the prepared inputs in this release may be passed to external metabolism models or to other model variants in the streamMetabolizer software), and (3) to draw attention to the potential of existing public datasets such as NWIS, NLDAS, and GLDAS for generating new information and insights.

## Methods

**Figure 1:**
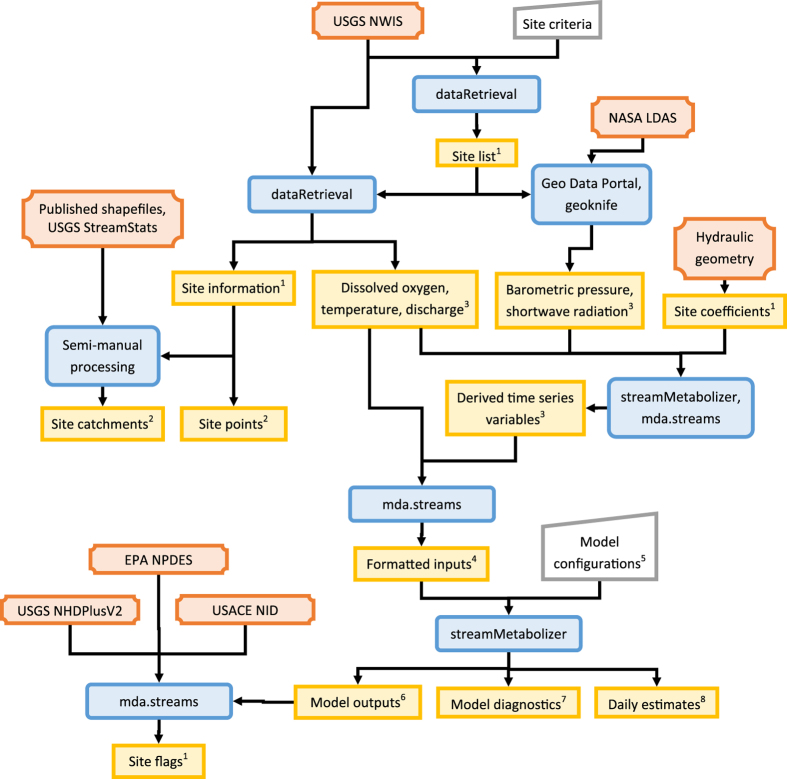
Inputs and workflow to generate metabolism estimates and supporting datasets. Inputs are either exogenous (dark orange plaque shapes) or encapsulate the authors’ configuration decisions (gray trapezoids). Data processing steps leverage several R packages and other tools (blue rounded rectangles); specifics of these steps are documented in the text. Data products included in this release (yellow rectangles) are organized into 8 final items (superscripts, corresponding to IDs in Table 1).

The preparation of this data release (Data Citation 1 and Table 1) involved multiple data sources and processing steps (Fig. 1): We identified sites amenable to the modeling approach, acquired site information and time series data from outside databases, derived additional forcing variables, selected those sites with all necessary data, configured and applied the metabolism estimation model, extracted model estimates, and collected and computed diagnostic metrics of model performance. We describe all these steps in detail below, beginning with an overview of the model.

### Metabolism model

We estimated metabolism by an open channel approach based on Odum’s classic method^9^, which relies on the fact that gross primary productivity (*GPP*), ecosystem respiration (*ER*), and physical air-water gas exchange are the dominant controls on the sub-daily dynamics of dissolved oxygen concentrations ([O_2_]), and these three processes can be differentiated because they each affect [O_2_] in different directions and with different timing. Our mass-balance-based approach fits modeled [O_2_] to observed [O_2_] to estimate the parameters *GPP*, *ER*, and a standardized rate coefficient for gas exchange (*K*600) using inverse modeling and Bayesian inference^10^. We estimated daily mean rates of metabolism and gas exchange for each site using the new streamMetabolizer software package^14,17^ in the R statistical programming language^18^. The streamMetabolizer package implements several model variants, so here we provide a brief overview of the specific variant named “b_Kb_oipi_tr_plrckm.stan”, which was the variant used for this analysis. Details of the model structure and statistical fitting procedure for this variant are in^14^, as is a discussion of alternative modeling approaches.

The core equation of the model gives the change in oxygen concentration at each timestep as:

(1)dOi,ddt=(GPPdz¯i,d×PPFDi,dPPFD¯d)+(ERdz¯i,d)+fi,d(K600d)(Osati,d−Oi,d)

where *O*_*i*,*d*_ is the modeled oxygen concentration on day *d* at time index *i*, and *dO*_*i*,*d*_/*dt* is a rate of concentration change. *GPP*_*d*_, *ER*_*d*_, and *K*600_*d*_ are the three daily parameters fitted by the model: *GPP*_*d*_ and *ER*_*d*_ are daily average rates of gross primary productivity and ecosystem respiration, respectively (g O_2_ m^−2^ d^−1^), while *K*600_*d*_ is a daily average value of the standardized gas exchange rate coefficient (d^−1^, scaled to a Schmidt number of 600). The other variables are model inputs: ${\bar{z}}_{i,d}$ is the stream depth (m) averaged over the width and length of the upstream reach; *PPFD*_*i*,*d*_ is the photosynthetic photon flux density (*μ*mol photons m^−2^ d^−1^); ${\overset{¯}{PPFD}}_{d}$ is the daily mean of observed *PPFD*_*i*,*d*_; *f*_*i*,*d*_(*K*600_*d*_) is a function that converts daily mean *K*600_*d*_ to an O_2_-specific, temperature-specific gas exchange coefficient (*KO*2_*i*,*d*_, d^−1^), and *Osat*_*i*,*d*_ is the theoretical saturation concentration of O_2_ if the water and air were in equilibrium.

Equation 1 is integrated using the trapezoid rule, as in^14^, to produce a time series of modeled [O_2_] to compare to the observed values. We chose a state space time series model so that we could incorporate both observation and process errors (i.e., fitting to match both the O_2_ concentrations and the stepwise concentration changes between observations). This method provides more accurate estimates of parameter values and parameter uncertainty than assuming either process error or observation error alone^14^.

We used a Bayesian Markov chain Monte Carlo (MCMC) fitting procedure to identify values of *GPP*, *ER*, *K*600, and several hierarchical parameters that balanced the model requirements to (a) produce a good match between observed and modeled [O_2_] and stepwise [O_2_] changes, and (b) stay consistent with our understanding of stream biology and physics. Specifically, we used partial pooling^19^ of *K*600 across all days in each site’s dataset, where daily values of *K*600_*d*_ were pooled toward a fitted, site-specific, piecewise linear relationship relating *K*600 to daily mean discharge, *Q*^14^. This relationship was built with many line segments to capture the potentially complex, idiosyncratic relationship at each site (see *Metabolism model configuration and application*). Each daily estimate *K*600_*d*_ was drawn from a normal distribution around the pooled prediction of *K*600 from the piecewise function of that day’s *Q*_*d*_. The standard deviation of that distribution was itself a fitted value drawn from a half-normal distribution. This partial pooling approach for *K*600 has been shown to reduce the number of extremely inaccurate estimates of *K*600, *GPP*, and *ER*, leading to greater accuracy overall^14^.

### Initial site selection

**Figure 2:**
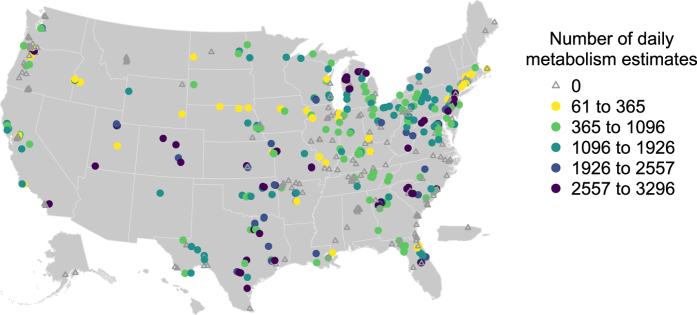
Sites included in this data publication. Sites that met the initial site selection criteria but did not have sufficient data to be modeled are gray triangles. Sites with sufficient data for modeling are filled circles, colored according to the number of dates for which estimates were produced (3296 days is 9.02 years).

We based our initial site selection on the availability of dissolved oxygen, the central variable necessary to model metabolism, in the USGS National Water Information System (NWIS, https://waterdata.usgs.gov/nwis)^20^. As such, the sites available in this dataset are limited to monitoring locations chosen for the purposes of other projects, resulting in non-uniform spatial and hydrologic coverage. In January 2017 we queried NWIS for stream and river monitoring sites with hourly or higher-frequency measurements of dissolved oxygen. To access NWIS we used the dataRetrieval software package^21^ in the R programing language^18^. We selected sites that were categorized as ST (stream), ST-CA (canal), ST-DCH (ditch), ST-TS (tidal stream), or SP (spring) and had at least 100 dissolved oxygen observations. This site list (Fig. 2) then formed the basis for acquisition of data from NWIS and other databases.

### Acquisition of site information

Data about each site were acquired both to support metabolism estimation and to provide context for interpreting the metabolism estimates; this information is included in “1. Site data” (Data Citation 1). We used the dataRetrieval software package^21^ to pull site information from the USGS NWIS database^20^ including the USGS site ID, a full site name, geographic coordinates, altitude, and the NWIS site type classification.

To facilitate cross-referencing this dataset with others, we associated each USGS site with a stream reach from the National Hydrography Dataset (NHDPlusV2, http://www.horizon-systems.com/NHDPlus, accessed April 2017)^22^. We identified the NHDPlusV2 reach code (ComID) for which the centerpoint latitude and longitude of that reach was closest to the latitude and longitude of the USGS site.

Hydraulic geometry coefficients were necessary to estimate depth and velocity at each site as functions of the reported discharge. Coefficients were obtained from a hydraulic geometry analysis^23^. The data used in that analysis were measurements of instantaneous low-flow and bankfull depths and widths made by the U.S. Environmental Protection Agency (EPA) at several thousand sites in the conterminous US^24,25^. Gomez-Velez *et al.*^23^ associated each EPA measurement site with the closest NHDPlusV2 reach and then correlated depths and widths with the cumulative drainage area reported in NHDPlusV2 to create standard power law relationships describing downstream changes in hydraulic geometry. The relationships were regionalized at the HUC2 (USGS Hydrologic Unit Code 2) level. Downstream hydraulic geometry equations were then developed for two flow frequencies, baseflow and bankfull conditions, which permitted at-a-station hydraulic geometry relationships to be developed by fitting power laws to depth and velocity as functions of discharge. The coefficients from^23^ were linked to the USGS sites in this metabolism analysis by NHDPlusV2 ComID.

For each site we collected two spatial features (2. Spatial data, Data Citation 1): the location of the site and the boundary of the contributing catchment. The site coordinates from NWIS were packaged into a single shapefile of site location points. Watershed boundaries were obtained from published sources or were delineated for this project (Table 2). Delineation was performed using StreamStats v.4.1.2 (https://streamstats.usgs.gov, accessed March 2017)^26^, a map-based web application from the U.S. Geological Survey that provides tools to delineate a drainage basin for a given latitude and longitude using stream flowlines from the National Hydrography Dataset (https://nhd.usgs.gov)^27^, the Watershed Boundary Dataset (https://nhd.usgs.gov/wbd.html)^28^, and elevation data from the USGS 3D Elevation Program (https://nationalmap.gov/3DEP)^29,30^. A total of 371 catchment boundaries were successfully obtained, describing all but 23 of the sites where metabolism was modeled and an additional 38 sites where dissolved oxygen data were available but metabolism was not ultimately modeled.

### Acquisition of timeseries observations

Several variables at hourly or finer temporal resolution are required to estimate metabolism. The direct model inputs to streamMetabolizer models are dissolved oxygen concentration, theoretical oxygen saturation concentration, stream depth averaged over the length and width of an upstream reach, water temperature, photosynthetic photon flux density, and discharge. We downloaded some of these variables directly from public databases (Table 3), while others were computed from variables in those databases (next section). Data for all timeseries variables are provided in “3. Timeseries data” (Data Citation 1).

Continuous time series data for dissolved oxygen, water temperature, and discharge were extracted from the USGS NWIS database^20^ using the dataRetrieval R package^21^ (Table 3). Downloads were limited to observations on or after October 1, 2007 for two main reasons: (a) high-frequency data from NWIS is not available through the public web interface before this date, and (b) earlier models of oxygen sensors were prone to lower precision and greater sensor drift because they relied on membrane rather than optical technology^31–33^. Temporal resolution of these timeseries data ranged from hourly to one observation every 5 minutes.

Data for atmospheric pressure and downward shortwave radiation flux were obtained for each site from NASA’s North American Land Data Assimilation System (NLDAS; http://ldas.gsfc.nasa.gov/nldas)^34,35^ and Global Land Data Assimilation System (GLDAS; https://ldas.gsfc.nasa.gov/gldas)^36^ (Table 3). These variables are available from NLDAS at hourly intervals and a 0.125° spatial resolution across continental North America from 25° to 53° North and −125° to −67° West, and from GLDAS at coarser temporal & spatial resolutions (3-hourly and 0.25°) but global spatial extent (thus including USGS sites in Alaska and Puerto Rico). Data were extracted from NLDAS and GLDAS at the selected USGS site locations using the geoknife R package^37^. GLDAS data were collected and are reported for all sites, but the higher-resolution NLDAS data were available and used in modeling all 356 sites that ultimately had the complete set of inputs necessary to estimate metabolism.

Data from both USGS NWIS and NASA NLDAS/GLDAS were pulled from those databases with timestamps already in UTC, thus avoiding the need to deal with variations in daylight savings time.

### Derivation of additional timeseries values

In addition to the 7 timeseries variables that could be downloaded directly from public databases (previous section), 18 additional variables were computed from those downloaded variables and other site information, both to directly support metabolism estimation and to provide context for interpreting metabolism estimates (Table 4).

Calculations relating to air pressure and saturation oxygen concentrations were implemented as functions within the streamMetabolizer package^17^. The calc_air_pressure() function was used to estimate the air pressure based on site elevation alone; the resulting variable, baro_calcElev, serves as a simpler alternative to baro_nldas and baro_gldas as downloaded from the NLDAS and GLDAS databases. The calc_DO_sat() function computes the theoretical concentration of oxygen if the air and water were at equilibrium, based on a function of water temperature, atmospheric pressure, and published coefficients^38,39^.

Calculations relating to time and light were also implemented in the streamMetabolizer package^17^. The convert_UTC_to_solartime() function converts clock times to solar times describing the position of the sun over a site. Solar time can take two forms, which are both included in this dataset and used for different purposes. Mean solar time (sitetime_calcLon and sitedate_calcLon), for which every day is exactly 24 hours but noon matches the sun’s zenith only approximately, is passed to the metabolism model and used only to determine the timestep length and assign each observation to a set of daily values of *GPP*, *ER*, and *K*600. Apparent solar time (suntime_calcLat), for which days are not exactly 24 hours but noon exactly corresponds to the sun’s zenith, is passed to the calc_light() function to model photosynthetic photon flux density above clouds (par_calcLat) based on the sun’s angle at a given latitude and apparent solar time. LDAS estimates of shortwave radiation are converted to photosynthetic photon flux density (par_calcSw) with a simple multiplier^40^ in the convert_SW_to_PAR() function. The calc_light_merged() function merges the modeled light from calc_light() with shortwave radiation data by multiplying modeled light by the linearly interpolated ratio of observed to modeled light, yielding a smooth interpolation from the hourly NLDAS or 3-hourly GLDAS data down to the finer temporal resolution of the [O_2_] data (usually at 5- to 30-minute intervals) (par_calcLatSw).

Calculations for other variables were implemented as simple function calls or equations in R; equations for these output variables are given directly in Table 4. Daily means and ranges were computed for the 24-hour windows from 4 a.m. to 3:59 a.m. to match the time windows used in estimating metabolism.

### Preparation of model inputs

The timeseries variables passed to the metabolism model were mean solar time (sitetime_calcLon), dissolved oxygen concentrations (doobs_nwis), saturation oxygen concentrations (dosat_calcGGbts), water temperature (wtr_nwis), PPFD (par_calcLatSw), discharge (disch_nwis), and stream depth as either depth_calcDischHarvey (347 sites) or depth_calcDischRaymond (9 sites) (Tables 3, 4). The data sources for each site are documented in “5. Model configurations” (Data Citation 1), and the resulting prepared model inputs are in “4. Model inputs” (Data Citation 1).

streamMetabolizer requires a single input table for each site, with one row per timestep and one column per timeseries variable. To create this merged table, we interpolated all non-[O_2_] variables to match the date-time values of the [O_2_] data, including filling any data gaps ≤3 h in length. If any variables had gaps >3 h, the entire 24-h period was excluded and no metabolism estimates were produced for that date. We used linear interpolation for most variables because linear interpolation of inputs generally yields similar metabolism estimates to interpolation by smoothing splines or other more complex methods^41^. However, we did use a more complex interpolation for light (PPFD), described above and yielding the calculated variable par_calcLatSw, to capture irregular fluctuations in light availability due to changing cloud cover.

Each metabolism model application could only accept a single temporal resolution of the input data, but some sites have varying temporal resolution of O_2_ observations over the course of the monitoring period (e.g., from hourly for several years to every 15 minutes for the remaining years). For such sites, we split the input data into one chunk for each temporal resolution. This led to 433 input datasets, and thus 433 model applications, for the 356 modeled sites.

### Site filtering

Only those sites with all necessary model inputs available concurrently could be modeled. 356 sites were retained in this filtering step.

### Metabolism model configuration and application

All model parameters are specified in the model configuration file in the data release (5. Model configurations, Data Citation 1). As in^14^, we used priors for *GPP* and *ER* based on the literature ranges described by Hall *et al.*^42^: normal priors were 3.1 (SD 6.0) g O_2_ m^−2^ d^−1^ for *GPP* and −7.1 (SD 7.1) g O_2_ m^−2^ d^−1^ for *ER*.

We fitted the pooled relationship between *K*600 and *Q* as a series of *N* linearly connected nodes at fixed intervals of 0.2 natural log units along the range of observed *Q*_*d*_ at each site. We used priors that encouraged the fitted *K*600_*n*_ value at each node *n* to be similar to those of adjacent nodes: the prior for log (*K*600_*n*_) was a normal distribution with standard deviation of 0.1 and mean equal to the value of the node to its left, log (*K*600_*n*−1_).

The prior on each daily *K*600_*d*_ value was a normal distribution centered on the corresponding prediction from the pooled *K*600∼*Q* relationship. The standard deviation of that normal distribution was itself a fitted value, shared across days and drawn from a half-normal prior distribution with mean 0 and standard deviation equal to 2% of of the median *K*600_*d*_ from a preliminary run of a model without pooling (streamMetabolizer model name “m_np_oi_tr_plrckm.stan”).

The model was applied using streamMetabolizer version 0.10.1 and R version 3.3.0 on Linux nodes in the HTCondor^43^ computing cluster at the USGS Wisconsin Water Science Center. Each model was initially run as four MCMC chains with 1000 warmup steps and 500 saved steps on each chain. Models that failed to converge with this number of iterations were re-run with 2000 warmup steps and 2000 saved steps. Individual models were run on 4 cores in parallel and required a median of 9.5 h and mean of 12.8 h per site-year of data, for a total of 17,168 h (715 d) of processing time. Because model runs were distributed over up to 300 cores at a time on the HTCondor cluster, the final batch run required roughly 3 wall-clock weeks.

### Preparation of model outputs

Internally, streamMetabolizer fits Bayesian models using the Stan software package^44,45^ and the rstan R interface to Stan^46^. Stan, and therefore streamMetabolizer, returns the following posterior distribution measures for every fitted parameter: mean, standard error, standard deviation, and the 2.5%, 25%, 50%, 75%, and 97.5% percentiles of the MCMC samples. For streamMetabolizer metabolism models, the fitted parameters include daily *GPP*_*d*_, *ER*_*d*_, and *K*600_*d*_; the *K*600_*n*_ values at nodes defining the *K*600∼*Q* relationship for each site, and overall model parameters including the standard deviations of [O_2_] observation error, process error, and deviations of daily *K*600_*d*_ from the pooled *K*600∼*Q* relationship. All distribution measures are reported for all parameters in “6. Model outputs” (Data Citation 1). In our streamlined table of daily *GPP*, *ER*, and *K*600 estimates for all sites (8. Metabolism estimates and predictors, Data Citation 1), we single out the 50% percentile value as the central estimate, and we report the 2.5% and 97.5% percentiles as bounds of the 95% credible interval.

Stan and streamMetabolizer also return two Bayesian model diagnostics for each parameter. The split R-hat statistic (Rhat, also known as the Gelman-Rubin convergence statistic), measures the consistency of the suite of the Markov chains with respect to a parameter^47^. The number of effective samples (n_eff) quantifies the estimation power of the Markov chains in terms of their equivalence to a number of independent samples, recognizing that each Markov chain sample is correlated with others and thus provides less new information than an independent sample^44^.

In addition to the above posterior distribution measures and model diagnostics, we also computed the following metrics for each model: the median *K*600_*d*_, the range of *K*600_*d*_ estimates between the 10% and 90% percentiles (to screen for physically unlikely variation in *K*600), the percent of *GPP* estimates <-0.5 and the percent of *ER* estimates > 0.5 (both are biologically unrealistic outcomes), and the number of hours that the model ran.

### Code Availability

For modeling we used version 0.10.1 of the streamMetabolizer package^14^. A snapshot of the package exactly as used for this analysis is at^48^. The development version of the package is at https://github.com/USGS-R/streamMetabolizer.

To support the activities of data acquisition, data preparation before modeling, preparation of the data release, and posting of data release files to the ScienceBase repository, we developed R scripts in the form of a project-specific package, mda.streams. A snapshot of the package as used for this analysis is at^49^. The development version of the package is at https://github.com/USGS-R/mda.streams.

Our complete workflow is documented as a collection of R scripts in a third repository, named stream_metab_usa, which makes use of the mda.streams and streamMetabolizer packages. This repository also makes heavy use of the remake R package^50^, which we used to orchestrate the flow of data and files through the many processing scripts. A snapshot of the scripts as used for this analysis is at^51^. The development version of the package is at https://github.com/USGS-CIDA/stream_metab_usa.

## Data Records

The data are stored in the USGS ScienceBase online data repository (Data Citation 1) and are organized into data items with titles “1. Site data” through “8. Metabolism estimates and predictors” (Table 1).

“1. Site data” provides USGS site information including the site ID, site name, coordinates and the coordinate datum, site altitude and the altitude datum, NWIS site type classification, and an associated NHDPlusV2 ComID. Six additional columns, prefixed by “dvqcoefs”, contain coefficients *c*, *f*, *a*, *b*, *k*, and *m* from the hydraulic geometry analysis^23^. Three final columns, prefixed by “struct”, provide site-level indicators of possible interference to metabolism estimation by infrastructure (canals, dams, and permitted waste discharge locations). This table combines information described in “Methods: Acquisition of site information” and “Technical Validation: Site suitability”.

“2a. Site coordinates” and “2b. Catchment boundaries” are two shapefiles containing site point locations and catchment boundary polygons, respectively. Each shapefile includes attributes for the NWIS site ID and the data source for spatial information. These files were prepared as described in “Methods: Acquisition of site information”.

“3. Timeseries data” provides timeseries data in separate files for each site and each variable listed in Tables 3 and 4 (i.e., both downloaded and computed variables). See “Methods: Acquisition of timeseries observations” and “Methods: Derivation of additional timeseries values”.

“4. Model inputs” also provides timeseries data, but in this case for a subset of variables as merged into one file per site and formatted for use in streamMetabolism models. See “Methods: Preparation of model inputs”.

“5. Model configurations” describes the configuration of each model application, including the provenance of the input data, the temporal resolution expected for the input data, and model priors and other specifications. This file was produced by a combination of scripted algorithms and human input, and it was directly ingested by the config_to_metab() function in our project-specific R package (mda.streams) to fit the metabolism models.

“6. Model outputs” reports the streamMetabolizer model outputs in detail at three levels per model: daily estimates of *GPP*_*d*_, *ER*_*d*_, and *K*600_*d*_; nodes defining the *K*600∼*Q* relationship, and overall model parameters. At each level we report all posterior distribution measures and model diagnostics produced by streamMetabolizer, for all parameters tracked by the model. Results are bundled into one zip file per model application. Daily estimates are indexed by date; nodes in the *K*600∼*Q* relationship are indexed by integers (4 to 75 nodes per model, median of 28), and overall model parameters only have one instance per model. See “Methods: Preparation of model outputs”.

“7. Model diagnostics” combines all high-level diagnostics into a single table with one row per model, including the model-level diagnostics reported by streamMetabolizer and the additional diagnostics computed as in “Methods: Preparation of model outputs”. This table also includes the results of our algorithm-based model assessment described below in “Technical Validation: Model performance”.

“8. Metabolism estimates and predictors” combines daily metabolism estimates into a single table for all sites, with one row per site-date combination. Values are reported for *GPP*, *ER*, and *K*600, and are limited for simplicity to the 2.5%, 50%, and 97.5% percentiles, Rhat, and n_eff for each parameter (see “Methods: Preparation of model outputs”). In addition to model outputs, this table includes 11 potential predictors of metabolic rates to facilitate further analyses of this dataset. These predictors are also included in “3. Timeseries data” (Tables 3 and 4) or are easily computed from those timeseries. Predictors include daily means of [O_2_], saturation oxygen concentrations, water temperature, shortwave radiation, stream depth, discharge, and velocity; the daily [O_2_] amplitude, the number of hours of daylight (PPFD > 0), and the 80% oxygen turnover distance. Sources for the first 10 predictors are as in Tables 3 and 4, while the turnover distance equation is in “Technical Validation: Site suitability”.

**Figure 3:**
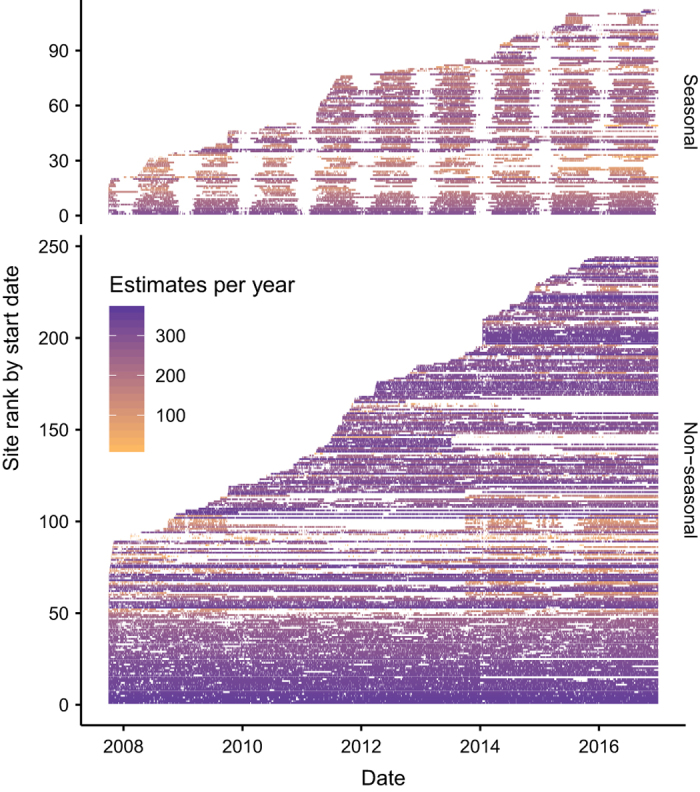
Temporal distribution of metabolism estimates at each site. Each site forms a row, and horizontal line segments represent periods of continuous daily metabolism estimates. Colors give density of estimates, ranging from 17 to 365 daily estimates per year. For the purpose of this figure, sites were considered “seasonal” if the number of metabolism estimates in January was fewer than 1/24 the total number of estimates at a site (112 of 356 sites meet this criterion).

Metabolism estimates and predictors are available for 356 sites (Fig. 2). Each site has between 61 and 3296 daily metabolism estimates, where 3296 dates is equivalent to just over 9 years (Fig. 3). The median density of these observations is 266 dates per year (range of 17 to 365 dates per year), with only 18 sites having fewer than 90 dates per year.

## Technical Validation

### Model requirements

All single-station metabolism models, including those used for this analysis, make inferences about metabolic activity in a stream reach extending upstream from the monitoring site. That upstream reach must meet several model assumptions to ensure accurate metabolism estimates^12,52,53^: (a) The reach must be well mixed in all dimensions, such that sensor observations describe the full stream reach accurately. (b) Rates of metabolism and gas exchange must be homogeneous throughout the reach. (c) Sources of oxygen to the reach must be limited to photosynthesis, gas exchange with the atmosphere, and water flowing from upstream. Sites are more likely to meet assumptions a and b when flow is unidirectional (not tidal or intermittent). Rapid variations in discharge and water sources, such as those occurring during storm onset, may violate assumptions b and c. To meet assumption c, the reach should also be free of groundwater inputs, hydrology-altering structures such as dams and canals, and [O_2_]-altering inputs such as wastewater.

Additionally, the accuracy of metabolism estimates depends on the presence of an [O_2_] signal that is strong enough to enable the model to distinguish among the [O_2_]-altering processes of photosynthesis, respiration, and gas exchange; these conditions are best met when *GPP* is high and *K*600 is low^7,14,54^.

We evaluated compliance with model requirements in three ways: we screened each day’s input data for evidence of violated assumptions, we looked for observable structural interferences, and we assessed the model output for signs of unrealistic predictions. Such assessments are especially important to this analysis because none of the input data were originally collected, nor site locations selected, for the express purpose of modeling metabolism.

Despite our substantial efforts to screen for unmet model requirements, these technical validation measures are neither exhaustive nor foolproof. Users should handle the model outputs with some skepticism and understanding that these estimates are only the best available, and likely imperfect, even for those dates, sites, and models for which no specific problems have been identified.

### Defining reach length

The length of the relevant upstream reach varies by site and over time because it is the distance over which the dissolved O_2_ pool undergoes near-complete turnover^55^. For the purposes of this data release, we defined the reach length on each day (*L*_*d*_, m) as the distance required for 80% gas renewal in the stream channel:

(2)Ld=−ln(1−0.8)×v/KO2d

where *v* is the daily average stream velocity (m d^−1^, computed as in “Derivation of additional timeseries values”) and *KO*2_*d*_ is the oxygen-specific gas exchange rate coefficient (d^−1^). *KO*2_*d*_ can be calculated from *K*600_*d*_ as

(3)KO2d=K600d×(ScO2/600)−0.5

where $Sc_{O_{2}}$ is the temperature-dependent Schmidt number^56^.

### Input data quality

We restricted our modeling to only those days with entirely positive flow, avoiding days of intermittent flow or tidal variation. Dates that did not meet this criterion are included in the prepared input data (4. Model inputs, Data Citation 1) but are excluded from the daily model estimates (8. Metabolism estimates and predictors, Data Citation 1). The tables of daily values in the detailed model output (6. Model outputs, Data Citation 1) contain messages explaining the exclusion of any dates that had some data but did not lead to a daily estimate.

We considered also excluding days with storm-driven discharge peaks, but preliminary inspection suggested that storm peaks do not always lead to unrealistic metabolism estimates. We have therefore left these dates in the dataset; however, we encourage users to detect and remove such days if the storm-day metabolism estimates appear to be unrealistic for the user’s target sites or if higher uncertainty about the accuracy of storm-day estimates cannot be tolerated.

Another aspect of input data quality that we cannot thoroughly assess is the accuracy of estimates of mean depth in the upstream reach. Mean depth is a direct scaling factor in the estimation of GPP and ER (Equation 1), such that metabolism estimates are sensitive to the depth value used. While we consider our approach for estimating mean depth to be highly data-rich and the best currently available at this national scale, we lack uncertainty information about these depths. As new datasets of stream depth become available in the future, we encourage users of our dataset to consider re-estimating metabolism with those new depth estimates and any accompanying uncertainty information, combined with the other input data already reported in our dataset. Improved characterization of reach depths should be an effective, if costly, next step in refining the metabolism estimates reported here.

### Site suitability

Our initial assessment of site suitability included visual inspection of discharge records for large diel variation in flow owing to tides, reservoir management, wastewater discharge, water withdrawal. We also inspected publicly available aerial imagery (https://www.google.com/maps) to identify impoundments and other structures. In the interests of transparency and reproducibility, we ultimately declined to exclude or flag sites on the basis of these assessments. However, we encourage users of this data to conduct similar qualitative assessments to ensure that sites meet the needs of their analyses.

We translated our visual inspection into a reproducible assessment with respect to the presence of dams, canals, and pollutant discharge points. Because the effects of such structures on metabolism estimates are variable and difficult to predict, we retained sites near such structures but provide indicators of the proximity of each site to those structures.

We gathered location data for structures of each type: dams in the National Inventory of Dams^57^; canals and ditches in the National Hydrography Database, Version 2 (NHDPlusV2, http://www.horizon-systems.com/NHDPlus, accessed November 16, 2015)^22^; and permitted point sources in the National Pollution Discharge Elimination System^58^. Spatial data layers for these features were clipped to watersheds upstream of the sites in our dataset. The geodetic distance between the site location and the nearest upstream feature of each type was then calculated using the function GenerateNearTable in the arcpy library in Python 3.6^59^.

The distances between sites and structural features were compared with the distributions of calculated reach lengths (80% O_2_ turnover distances, Equation 2). The structure indicators in “1. Site data” (Data Citation 1) specify whether a structure was located beyond the 0th, 50th, 80th, or 95th percentile of daily reach lengths at a site. These correspond to fewer than 0%, 50%, 20%, and 5% of the modeled days having <80% gas turnover between the upstream feature and the probe, respectively (Table 5).

A high value of a structural interference metric on a site or date is no guarantee that all model requirements have been met. In particular, site metrics are unavailable where catchment shapefiles were unavailable (23 sites), and we lack data on all possible interferences; others could include dams too small to be documented in the National Inventory of Dams, heterogeneity in stream habitat or riparian shading within the upstream reach, or natural inputs of O_2_-depleted groundwater.

### Model performance

To summarize numerous metrics of model performance, we developed an algorithm to label each model as likely deserving of Low, Medium, or High confidence. This model assessment is based only on readily computable information and is intended only as guidance, not an incontrovertible evaluation, such that an expert familiar with a site or its oxygen patterns could reasonably override the assessment given here. To support customized assessments, we also provide site information (1. Site data, Data Citation 1), raw data for [O_2_] and other variables (“3. Timeseries data” and “4. Model inputs”, Data Citation 1), and Rhat and n_eff values for all fitted parameters (6. Model outputs, Data Citation 1).

Our summary model assessment was based on model convergence, variation in predicted *K*600, and presence of biologically unrealistic *GPP* and *ER* values (Table 6). We inferred problems with overall convergence when Rhat >1.2 for either of two key parameters: the standard deviation of *K*600_*d*_ deviations from the pooled *K*600∼*Q* relationship (${\hat{R}}_{SD(K600)}$) and the standard deviation of process error (${\hat{R}}_{SD(\varepsilon_{p})}$). We also considered the difference in *K*600_*d*_ estimates between the 10th and 90th quantiles (P_90_–P_10_), which we interpreted as unrealistic when >50 because *K*600 is constrained by channel shape so should not vary dramatically within a site; we gave the highest ratings to models for which P_90_–P_10_<15. Finally, because it is biologically unrealistic for *GPP* to be negative or *ER* positive, we computed the percentages of *GPP*_*d*_ estimates below −0.5 and *ER*_*d*_ values above 0.5, assuming values between -0.5 and 0.5 are difficult to distinguish from 0. We assigned Low confidence when >50% of *GPP*_*d*_ values were <−0.5 or >50% of *ER*_*d*_ values were >0.5, Medium confidence when 25%–50% were beyond these thresholds, and High when <25% were beyond these thresholds. Although we treated *GPP*_*d*_ and *ER*_*d*_ similarly, note that when models are classified as Medium or Low confidence only because of positive *ER*_*d*_, the *GPP*_*d*_ estimates are often still reliable: positive *ER*_*d*_ often reflects a miscalibrated oxygen sensor, which does not affect *GPP*_*d*_ estimates because those are based on [O_2_] changes rather than absolute values.

Assessments of each model were summarized at the site level (recall that some sites had multiple models) in the form of (a) a minimum site confidence and (b) a comma-separated list of all model confidence values that were assigned to models for that site (7. Model diagnostics, Data Citation 1).

## Usage Notes

The approach taken in this modeling effort emphasizes breadth over precision, in that modeling was attempted for the largest feasible number of sites and days. Some sites and days for which model estimates are reported likely have inaccurate estimates. Analyses using these model estimates will vary in their requirements for the accuracy of metabolism estimates, so we report the largest possible number of estimates with the expectation that most data users will filter this complete dataset down to the estimates that are appropriate for their analysis. For example, analyses of seasonal patterns or annual averages across many sites may be more forgiving than comparisons of a specific pair of sites on individual days.

## Additional information

**How to cite this article**: Appling, A. P. *et al*. The metabolic regimes of 356 rivers in the United States. *Sci. Data*. 5:180292 doi: 10.1038/sdata.2018.292 (2018).

**Publisher’s note**: Springer Nature remains neutral with regard to jurisdictional claims in published maps and institutional affiliations.

## Supplementary Material



## Figures and Tables

**Table 1:** Data items included in 1.

ID	Title	Description	Format
1	Site data	Site identifiers, details, and quality indicators	Table with 1 row per site (tab-delimited file)
2	Spatial data	Site coordinates (2a) and catchment boundaries (2b)	1 shapefile for all coordinates and 1 for all catchments (.shp, .shx, .dbf, and .prj files)
3	Timeseries data	Data on water quality and quantity, collected or computed from outside sources	Tables with one row per time series observation (1 tab-delimited file per site-variable combination, 1 zip file per site)
4	Model inputs	Data formatted for use in estimating metabolism	Tables of prepared time series inputs (1 tab-delimited file per site, in 1 zip file per site)
5	Model configurations	Model specifications used to estimate metabolism	Table with 1 row per model (1 tab-delimited file, compressed into zip file)
6	Model outputs	Complete fits from metabolism estimation models	Text and 4 tables for each model (tab-delimited files, 1 zip file per model)
7	Model diagnostics	Key diagnostics and overall assessments of model performance	Table with 1 row per model (1 tab-delimited file, compressed into zip file)
8	Metabolism estimates and predictors	Daily metabolism estimates and potential predictor variables to support further exploration	Table with 1 row per site-date combination (1 tab-delimited file, compressed into zip file)

**Table 2:** Data sources for boundaries of the catchments contributing to sites in this data release.

Description	Reference	Number of Basins
EPA BASINS	^60^	262
USGS StreamStats	^26^	54
USGS GAGES-II	^61^	27
Falcone *et al.* 2017	^62^	11
Wieczorek 2012	^63^	9
USGS National Map Viewer	^64^	7
Nakagaki *et al.* 2016	^65^	1

**Table 3:** Definitions and provenance of timeseries variables downloaded from external databases.

Variable Name	Description (Units)	Source Database	Parameter Code
disch_nwis	Discharge (ft^3^ s^−1^)	^20^	00060
doobs_nwis	Dissolved oxygen concentration (mg O_2_ L^−1^)	^20^	00300
wtr_nwis	Water temperature (°C)	^20^	00010
baro_nldas	Surface pressure (Pa)	^34,35^	pressfc
baro_gldas	Surface air pressure (Pa)	^36^	psurf_f_inst
sw_nldas	Downwards shortwave radiation flux, surface (W m^−2^)	^34,35^	dswrfsfc
sw_gldas	Downward shortwave radiation flux, surface (W m^−2^)	^36^	SWdown_f_tavg

**Table 4:** Definitions and provenance of calculated timeseries variables.

Variable Name	Description (Units)	Sources	Equation or streamMetabolizer function
baro_calcElev	Surface pressure (Pa)	altitude	calc_air_pressure()
depth_calcDischHarvey	Stream depth (m)	*c* and *f* ^23^, disch_nwis	*c*×disch_nwis^*f*^
depth_calcDischRaymond	Stream depth (m)	*c* and *f* ^56^, disch_nwis	*c*×disch_nwis^*f*^
dischdaily_calcDMean	Daily average discharge (m^3^ s^−1^)	disch_nwis	Daily mean (4am-3:59am)
doamp_calcDAmp	Daily amplitude in percent O_2_ saturation (%)	dopsat_calcObsSat	Daily range (4am-3:59am)
dopsat_calcObsSat	Percent O_2_ saturation (%)	doobs_nwis, dosat_calcGGbts	100×doobs_nwis/dosat_calcGGbts
dosat_calcGGbconst	[O^2^] at saturation (mgO_2_ L^−1^)	baro_calcElev	calc_DO_sat()
dosat_calcGGbts	[O_2_] at saturation (mgO_2_ L^−1^)	baro_nldas or baro_gldas	calc_DO_sat()
par_calcLat	Photosynthetic photon flux density, PPFD (*μ* mol m^−2^ s^−1^)	suntime_calcLon, latitude	calc_light()
par_calcLatSw	PPFD (*μ* mol m^−2^ s^−1^)	par_calcLat, par_calcSw	calc_light_merged()
par_calcSw	PPFD (*μ* mol m^−2^ s^−1^)	sw_nldas or sw_gldas	convert_PAR_to_SW()
sitedate_calcLon	Solar noon of the date (unitless)	DateTime	convert_UTC_to-_solartime()
sitetime_calcLon	Mean solar time (unitless)	DateTime, longitude	convert_UTC_to-_solartime()
suntime_calcLon	Apparent solar time (unitless)	DateTime, coordinates	convert_UTC_to-_solartime()
swdaily_calcDMean	Daily average downwards shortwave radiation flux (W m^−2^)	sw_nldas or sw_gldas	Daily mean (4am-3:59am)
veloc_calcDischHarvey	Stream velocity (m s^−1^)	*k* and *m*^23^, disch_nwis	*k*×disch_nwis^*m*^
veloc_calcDischRaymond	Stream velocity (m s^−1^)	*k* and *m*^56^, disch_nwis	*k*×disch_nwis^*m*^
velocdaily_calcDMean	Daily average velocity (m s^−1^)	veloc_calcDischHarvey or veloc_calcDischRaymond	Daily mean (4am-3:59am)
Sources include other variables from this table, DateTimes of the [O_2_] data, hydraulic geometry coefficients from the cited sources, and site data (altitude, latitude, longitude). Where Sources are “X or Y”, the source ending in _nldas was preferred over _gldas, and _calcDischHarvey over _calcDischRaymond, whenever available.			

**Table 5:** Counts of sites by distance to nearest structure, for the 333 modeled sites with catchment information.

Structure	P_0_	P_50_	P_80_	P_95_
Canal/ditch	61	13	13	246
Dam	169	29	30	105
NPDES	130	38	27	138
Any	210	32	27	64
Column names give the lower bound on the distance, as a percentile of each site’s daily reach lengths, to the nearest structure of each type. For example, the nearest canal or ditch is located between the 0th and 50th percentile of reach lengths at 61 modeled sites; the nearest canal or ditch is beyond the 95th percentile at 246 sites; and 64 sites have no known structure closer than the 95th percentile of their reach lengths.				

**Table 6:** Model output assessment criteria and counts of models meeting each criterion.

Measure	Low	Medium	High
max(${\hat{R}}_{SD(K600)}$, ${\hat{R}}_{SD(\varepsilon_{p})}$)	>1.2 (49)	n.a.	<1.2 (384)
*K*600 range (P_90_ −; P_10_, d^−1^)	>50 (7)	15–50 (52)	<15 (374)
Negative GPP (%)	>50 (5)	25–50 (4)	<25 (424)
Positive ER (%)	>50 (17)	25–50 (35)	<25 (381)
Overall confidence	71	63	299
Meeting any of the criteria in the Low column earns a model Low confidence, and meeting all criteria in the High column is required to earn High confidence. Parentheses in the table body contain the number of models meeting each criterion.			
